# Economic burden of drug overdose deaths before and during the coronavirus disease 2019 pandemic in the USA

**DOI:** 10.2217/cer-2021-0314

**Published:** 2022-05-06

**Authors:** Briana Lui, Robert S White, Jaime Aaronson, Marguerite Hoyler

**Affiliations:** ^1^Department of Anesthesiology, Weill Cornell Medicine, 525 East 68th Street, Box 124, New York, NY 10065, USA; ^2^Department of Anesthesiology, New York Presbyterian Hospital/Columbia University Irving Medical Center, 622 West 168th Street, PH 5-505, New York, NY 10032, USA

**Keywords:** COVID-19, drug overdose, health economics

## Abstract

**Aim:** To evaluate the impact of the coronavirus disease 2019 pandemic on the economic burden of drug overdose deaths in the USA. **Methods:** Overdose death counts from 2019 to 2020 were obtained from the CDC's National Vital Statistics System. Years of potential life lost and value of statistical life were computed. **Results:** The financial burden of overdose deaths increased by nearly 30%, from US$624.90 billion before the pandemic in 2019 to US$825.31 billion during the pandemic in 2020. Temporal analysis demonstrated that overdose deaths peaked in the second quarter of 2020 and contributed to nearly a third of the total 2020 value of statistical life. **Conclusion:** The authors' findings suggest that the coronavirus disease 2019 pandemic has exacerbated the US drug overdose epidemic.

Since the start of the coronavirus disease 2019 (COVID-19) pandemic in 2020, data from the national, state and local levels have shown a substantial uptick in drug overdose deaths across the USA. In November 2021, preliminary data from the CDC projected a 28.5% increase in drug overdose deaths over a 12-month period ending in April 2021, with an estimated total of 100,306 deaths [1]. This drastic surge in overdose deaths has been attributed to the disruptions in employment, healthcare and community and social services caused by the pandemic [2–4], which have resulted in over 820,000 deaths across the nation as of December 2021 [5]. Studies have shown that the financial and social stressors of the pandemic have had significant adverse mental health consequences, including pervasive uncontrolled fears related to infection, anxiety, frustration and loneliness [2]. These psychological effects may disproportionately affect vulnerable populations, such as patients with existing or a history of substance use disorders and/or mental health conditions, increasing the risk of drug usage and accidental or intentional overdose deaths [3]. Moreover, the pandemic has disrupted illicit drug pipelines, potentially resulting in contaminated and more lethal drugs, and has reduced access to evidence-based treatment, harm reduction services and emergency medical care, all of which may contribute to the increase in overdose-related mortality [4].

As US public health agencies grapple with the concurrent crises of COVID-19 and the drug overdose epidemic, monitoring the impact of the COVID-19 pandemic on drug overdose deaths is necessary to inform an effective public health response and to mitigate further loss of life. Although prior studies have examined temporal patterns in overdose-related emergency medicine encounters [6,7] and overdose death rates [8–10] at a state level since the start of the pandemic, they have yet to quantify the fiscal burden of drug overdose deaths before and during the pandemic on a national scale. Cost–benefit metrics such as years of potential life lost (YPLL) and value of statistical life (VSL) are useful tools for assessing the financial burden of lives lost and are commonly used by policymakers to estimate values of risk reduction strategies when considering new health regulations and safety measures [11]. This study aims to evaluate the impact of the COVID-19 pandemic on the economic burden of premature deaths due to drug overdose in the USA, as measured by YPLL and VSL.

## Methods

### Study databases & population

Drug overdose death counts in the USA by quarter (Q1 2019 through Q4 2020), sex, age, race and Hispanic origin were obtained from the CDC's National Vital Statistics System [12]. Data for 2019 were based on final data, and data for 2020 were provisional and subject to change. Death counts included drug overdose deaths that occurred in the USA among US residents and were identified using *International Classification of Diseases, Tenth Revision, Clinical Modification* codes X40-44 (unintentional), X60-64 (suicide), X85 (assault) and Y10-14 (undetermined intent).

Period life expectancies for males and females aged 0–119 years were obtained from the 2019 Social Security actuarial life table [13]. Period life expectancy at a given age was defined as the average remaining number of years expected prior to death for a person at that exact age, born on January 1, using the mortality rates for 2019 over the course of his or her remaining life. The Social Security actuarial life table is based on the mortality experience of a population composed of residents of all 50 states and the District of Columbia (adjusted for net census undercount); civilian residents of Puerto Rico, the Virgin Islands, Guam, American Samoa and the Northern Mariana Islands; federal civilian employees and persons in the US Armed Forces abroad and their dependents; non-citizens living abroad who are insured for Social Security benefits; and all other US citizens abroad. This study used publicly available data and did not require institutional review board approval.

### Statistical analysis

The upper value of the midpoint of each age range was used to determine baseline age of death. For instance, the baseline age of death for an age range of 15–24 years was 20. The age ranges provided by the National Vital Statistics System were 0–14 years, 15–24 years, 25–34 years, 35–44 years, 45–54 years, 55–64 years, 65–74 years, 75–84 years, 85 years and over and not stated. For 85 years and over, the age range was set at 85–94 years, with a baseline age of death of 90. Deaths with unknown ages (n = 21) were excluded from analysis.

The primary outcomes of this study were total YPLL and total VSL due to drug overdose from Q1 2019 to Q4 2020. Quarterly YPLL for each age range was determined by aggregating the product of the male life expectancy at the baseline age of death and the proportion of overdose deaths that were male with the product of the female life expectancy at the baseline age of death and the proportion of overdose deaths that were female. Quarterly VSL for each age range was determined by multiplying YPLL by the population average VSL year, which was set at US$240,676 based on prior literature [14]. Total YPLL and total VSL for each quarter from 2019 to 2020 were computed by aggregating YPLL and VSL for each age range. Sensitivity analysis was performed using the upper and lower bound of each age range to determine the range of YPLL and VSL.

## Results

The total number of drug overdose deaths nationwide per year increased from 70,630 deaths in 2019 to 91,773 deaths in 2020. Males made up over two-thirds of all overdose-related deaths. The majority of drug overdose victims were non-Hispanic Whites followed by non-Hispanic Blacks and Hispanics.

**Figure 1.**
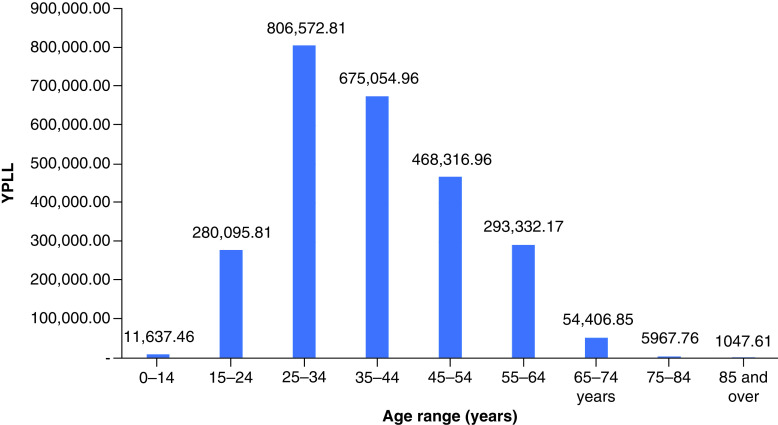
Years of potential life lost by age range in 2019. YPLL: Years of potential life lost.

**Figure 2.**
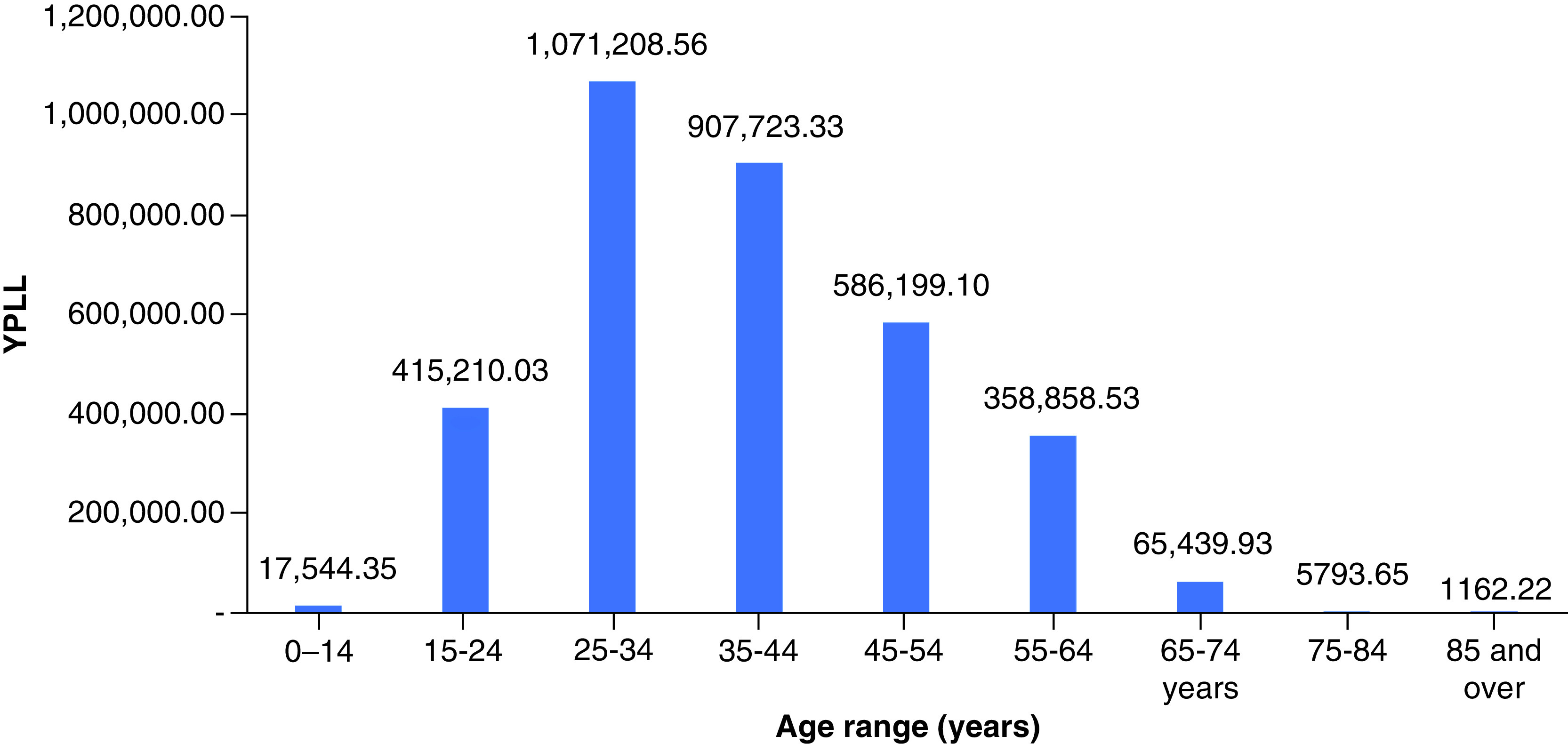
Years of potential life lost by age range in 2020. YPLL: Years of potential life lost.

**Figure 3.**
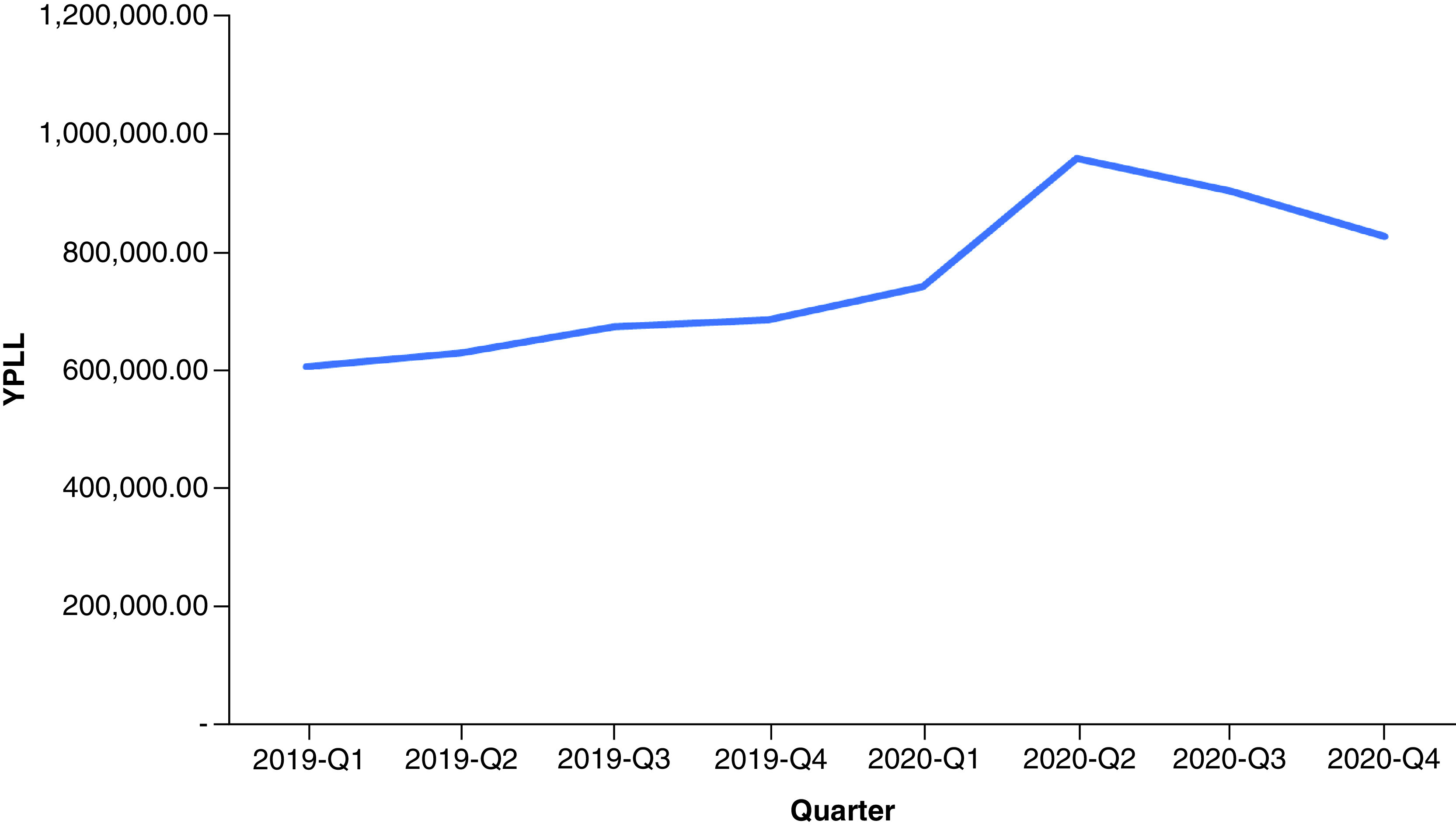
Years of potential life lost by quarter from 2019 to 2020. YPLL: Years of potential life lost.

**Figure 4.**
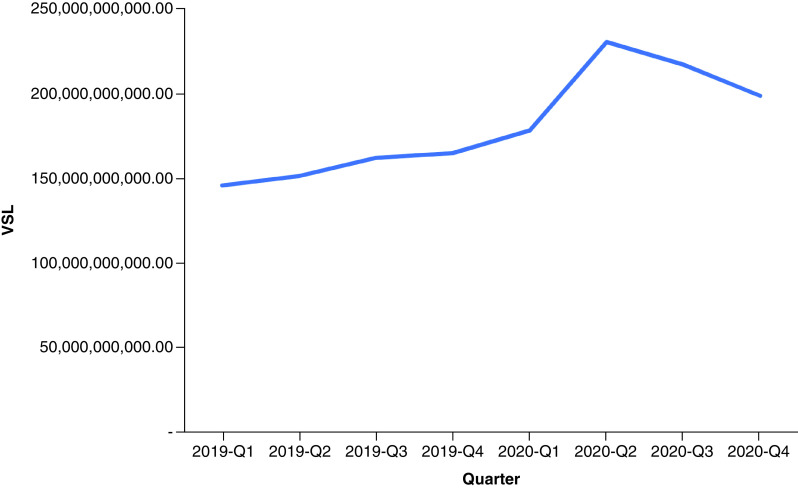
Value of statistical life by quarter from 2019 to 2020. VSL: Value of statistical life.

In 2019, drug overdose mortality resulted in a total YPLL of 2,596,432 (range: 2,352,500–2,826,573) and a total VSL of US$624.90 billion (range: US$566.19–680.29 billion) (Table 1). Total YPLL and VSL rose by nearly 30% to 3,429,140 (range: 3,110,807–3,836,537) and US$825.31 billion (range: US$748.70–923.36 billion), respectively, in 2020. Overdose deaths in the 25–34 and 35–44 age ranges contributed to roughly 60% of the total YPLL in both 2019 and 2020 (Figures 1 & 2). Temporal analysis by quarter showed an upward trend in YPLL and VSL from Q1 2019 (YPLL: 606,475; VSL: US$145.96 billion), increasing by approximately 22% in Q1 2020 to YPLL of 741,402 and VSL of US$178.44 billion and by 58% in Q2 2020 to YPLL of 958,430 and VSL of US$230.67 billion (Figures 3 & 4). The economic burden of lives lost to drug overdose decreased slightly in the last two quarters of 2020, although quarterly YPLL and VSL were still greater than that observed in 2019.

**Table 1.** Total years of potential life lost and total value of statistical life by year.

Deaths	2019		2020	
	70,630		91,773	
	Total YPLL	Total VSL, USD	Total YPLL	Total VSL, USD
Base case	2,596,432	624,898,958,538	3,429,140	825,311,624,353
Lower bound	2,352,500	566,190,280,520	3,110,807	748,696,531,361
Upper bound	2,826,573	680,288,278,159	3,836,537	923,362,439,029

USD: US dollars; VSL: Value of statistical life; YPLL: Years of potential life lost.

## Discussion

This study assessed the relationship between the onset of the COVID-19 pandemic and the economic burden of drug overdose mortality from 2019 to 2020, as measured by the cost–benefit metrics YPLL and VSL. Estimations of VSL represent the amount of additional spending that would be commonly accepted as justifiable to prevent excess mortality due to opioid overdose and may guide policymakers in estimating the cost of enhanced public health safety measures.

Results demonstrated a drastic surge in the financial consequences of overdose deaths over a 1-year period, increasing by nearly 30%, from US$624.90 billion before the pandemic in 2019 to US$825.31 billion during the pandemic in 2020. Quarterly trends suggested that drug overdose deaths were already on the rise prior to the pandemic, consistent with early public health reports documenting a worsening drug overdose epidemic involving potent synthetic drugs often in combination with other substances [15]. Temporal analysis by quarter further showed that the economic burden of lives lost to drug overdose peaked in Q2 2020 and contributed to nearly a third of the total VSL in 2020; this correlates with a time point when health systems across the nation were stretched beyond capacity and state government officials issued stay-at-home mandates to prevent the spread of the virus. Although results demonstrated a decrease in the cost of premature deaths due to drug overdose in the last two quarters of 2020, this is likely an underestimate given that the obtained 2020 data were provisional and subject to change.

There are several limitations to consider in this study, including the use of VSL, which approximates an individual's willingness to pay for risk reduction and may be inadequate for analyzing policies that mitigate large-scale risks such as COVID-19 and drug overdose [14]. Moreover, VSL was determined from a set VSL year that did not account for heterogeneity in age, socioeconomic status and race and ethnicity [14,16]. Overdose death data from the National Vital Statistics System did not include information on type of drug overdose (e.g., opioid, methadone, heroin), income level, race and ethnicity or existing health conditions (e.g., substance use disorder, mental health conditions) stratified by age group. Such information would be helpful in assessing potential variations in fiscal burden and targeting cost–benefit interventions to specific populations to reduce mortality risk.

## Conclusion

The COVID-19 pandemic has exacerbated the drug overdose epidemic in the USA and may have long-term negative implications on overdose mortality. Our study demonstrated a substantial increase in YPLL and VSL due to drug overdose nationwide from before the pandemic to during the pandemic.

As new and more potent variants of the virus emerge [17], the risk of drug overdose will likely increase as a result of a complex combination of factors, including financial and social stressors, disruption in drug supply and reduced access to harm reduction services. Mitigating the economic burden of drug overdose mortality will require unprecedented efforts and collaboration on a national, state and local level to provide support and broaden treatment access to those at risk of drug overdose. Findings from this analysis can inform public health officials and researchers on the value of risk reduction strategies, including new public health policies and outreach programs aimed at preventing opioid overdose mortality.

**Summary points**

- The estimated years of potential life lost and value of statistical life due to drug overdose in the USA have increased substantially since the start of the coronavirus disease 2019 pandemic.
- The economic burden of lives lost increased by nearly 30%, from US$624.90 billion before the pandemic in 2019 to US$825.31 billion during the pandemic in 2020.
- Temporal analysis demonstrated that overdose deaths peaked in the second quarter of 2020 and contributed to nearly a third of the total 2020 value of statistical life.
